# Martin’s Bandages

**Published:** 1879-08

**Authors:** Henry A. Martin

**Affiliations:** 27 Dudley St., Boston


### ARTICLE XIII.

### A LETTER FROM DR. HENRY A. MARTIN.

*Messrs. Editors:*

The "New York Letter" in your journal for May contains the following, "The gum bandage is being used very extensively; in synovitis, arthritis, in sprains and bruises it seems to act remarkably well. Its magical effect on old ulcers is not so prominent. The ordinary treatment for these is to first wash them off with carbolized water, 1 to 20, then cover them with cotton that has been soaked in a saturated solution of boracic acid; over this the gum bandage is applied. Under this treatment the ulcers soon fill up with granulations, but cicatrization is by no means rapid, and it seems to be thought by the staff that those who have secured such wonderful results with this bandage have mistaken granulation for cicatrization."

As the first printed publication of the method of treatment, not only of ulcers of the leg, but of a very extensive and various series of other diseases and injuries, principally though not exclusively of the lower extremities, by the application of bandages of "pure rubber" was made by myself in the issue of your valuable journal for November, 1877, and as my exclusive claim to the invention of that method has, marvellous to relate, not been questioned, I am naturally interested in whatever appears in *your* pages on the subject, and I hope excusable for offering some comments suggested by the above quotation.

Whatever the merits of the method of treatment of ulcers at the Roosevelt Hospital, it is not *my* method. It is doubtless considered an *improvement* upon it. In every publication or other utterance that I have ever made on the subject I clearly recommend that a bandage of what is technically called "pure rubber" shall be applied *directly* to the skin and ulcerated or otherwise diseased surface, *without any other dressing whatsoever.* To the application and use of such a bandage, pure and simple, my own long and large experience, now confirmed by that of many thousand surgeons, not only in America but, and more numerously, in Europe, has led me to confidently ascribe an efficacy never before even distantly approached, in large classes of cases, in which are included vast numbers of instances of the most miserable and loathsome, though not lethal, of human physical infirmity; in which, indeed, all previous treatment had been extremely irksome and laborious to the practitioner, tedious and expensive to the patient, while its results were often so long postponed as to leave considerable doubt in the minds of both parties whether they were results of treatment and not of more or less impeded reparative effects of nature only; and, too often, these results were far from complete and enduring. I have not neglected the use of anything under the bandage from a lack of knowledge, or, at any rate, of experience of the value or merit of the means with which, not only the "surgical staff of the Roosevelt Hospital," but several other practitioners also, have seen fit to complicate and "improve" my method.

In the paper in which (*British Medical Journal*, Oct. 26th, 1878) I asked the attention of the profession of Great Britian to the use of the pure rubber bandage, I wrote:

"It" (the bandage) "is to be applied directly to the skin and surface of the ulceration without any lotion, salve, or other dressing whatsoever. The statement attributed to Mr. Hutchinson, that a piece of lint may be placed over the ulcer, has no authority from me. An essential point in the method is that *nothing whatever* shall be applied between the bandage and the ulcer. In a very few cases, a hyperæsthetic patient has been, or thought he (or she) was unable at first or at all to endure the rubber next the skin, and, in these cases (not one in a hundred), I have directed that a linen, cotton, or thin flannel "roller" should be applied to prevent the immediate contact of the rubber. I have done this rather than renounce the great advantages of the india-rubber bandage entirely; *but it always delays the cure of the ulcer.* In cases of uncomplicated varicose disease, or in many joint cases in which the important use of my bandage is the support and security it affords, there is no objection to the application of an ordinary bandage under the rubber; but in cases of ulcer, eczema, and other cutaneous diseases, and many where the joints are affected, it is necessary, for the full attainment of the benefits of the treatment, that the bandage should be in immediate and close contact with the *skin.*"

In the other two principal papers which I have published on the subject, I have, in other words, expressed precisely the same opinions. These plainly expressed notions of mine do not, of course, prove that the Roosevelt Hospital method is not much better, but they sufficiently prove—and that is my present object—that my method differs very essentially from that of the New York surgeons, and if the reader will take the trouble to refer to the papers to which I have alluded, he will find that before I reached the perfect simplicity of treatment which I practice and commend, I had thoroughly and patiently tested and abandoned entirely the Roosevelt method, and many more or less like it. I in no way criticise the New York "*improvement*," or deny that it is one, except so far as to say that in my opinion the application of *anything* between a properly made bandage and the skin will fail to promote, in the slightest degree, the ends attained by the proper application of the bandages *alone*, while carbolic or boracic acid, whether an ingredient of the bandage (as the former is of many advertised and sold in large numbers as "Martin's bandages"), or in ointment or lotion, alone or saturating a compress applied to a wound or ulcer, unless in an extremely dilute state, will very much delay the process of cicatrization. Lister's antiseptic method avoids, with the most elaborate and complicated care, the contact of carbolic or boracic acid with granulating surfaces. That great theorist is also enough of a surgeon to know that the *immediate* contact of such substances very seriously interferes with the process of cicatrization. In many hospitals, however, in America, the antiseptic method, an "improvement" on Lister's, consists in the most liberal inundation of wounds, ulcers, compound fractures—

everything—with the inevitable carbolized solution or oil. So much is this method esteemed, that it seems to take the place of and to be supposed to obviate the necessity for many antiquated notions of pre-carbolic surgery, as counter-openings, drainage, etc., etc. Your New York correspondent's quotation of the sage surmise that gentlemen who had reported ulcers rapidly and solidly healed by "Martin's bandage" did not know what they were talking about, and mistook granulation for cicatrization, is very far from complimentary to a very large and very illustrious body of practitioners in Europe and America. Do the surgical staff of the Roosevelt Hospital really think that there is a practitioner anywhere so ignorant as not to know an ulcer healed from an open ulcer. If that distinguished staff, in its same self-complacency and associated contempt for almost everybody else, is so credulous, does its credulity extend to the belief that Prof. Gunn, of Chicago, their own townsman Dr. L. D. Bulkley, Mr. G. W. Callender (Surgeon of St. Bartholomew's), and Jonathan Hutchinson, one of the very greatest surgeons living, and many equally eminent, who have all reported large numbers of cases of ulcer healed by the use of "Martin's bandage" *alone*, without any of the Roosevelt improvements, are also ignorant of the difference between cicatrization and granulation; that, in a word, that subtle and sublime item of surgical wisdom is well-nigh exclusively the prerogative of the "surgical staff of Roosevelt Hospital?"

I wish to say something more in regard to "gum" bandages. The use of the simple word "gum" for india-rubber and for all its compounds was, I think, principally confined to New York, and little known to the world outside of that favored city, till certain scandalous passages in its social history brought into the secular press of the country a racy correspondence touching a certain pair of "gum shoes." The material of which gum shoes are made contains a very small percentage of gum caoutchouc. Very few of the almost innumerable articles known as "rubber" contain more than a small proportion of india rubber, their ingredients, besides this, being very numerous and various. Carbonate of lead, oxide of zinc, oxide of mercury, sulphuret of antimony, sulphide of carbon, whiting, carbolic acid, litharge, wood naphtha, and an almost interminable list more. Bandages made of "gum" or "rubber," containing such substances as carbolic acid, sulphide of carbon, or carbonate of lead are totally unfit to be applied for any length of time to a sound cutaneous surface, and much less to an ulcerated or otherwise diseased or wounded one. If the bandages used at Roosevelt Hospital are like many I have seen, the method of preventing their contact with the skin is very wise and proper.

Immediately after the publication of my method, in Europe and in this country, there arose a very great demand for "Martin's bandages." To meet that demand, in obedience to a well known law, manufacturers produced immense quantities of bandages totally unfit for use, worthless or nearly worthless from their want of durability, and worse than worthless, absolutely injurious as applications, as I have directed, immediately and for long periods to the surface of the body. Of all these bandages, of which I have seen specimens, there was but one which I could conscientiously approve, and even this would prove unsatisfactory on the score of durability, for the rubber of which it was made, though of excellent quality was "green," *i. e.*, quite unseasoned. Not one of the rubber manufacturers ever came or wrote to me for directions or suggestion or even apparently (save in one instance) thought it worth while to obtain one of my bandages as a pattern, and so, without any knowledge or appreciation of the importance of making them of certain material and in a certain way, they produced and sold thousands of bandages, reports of the want of durability of which, and of the imperfect and unsatisfactory results from the use of which are made by men who I know in some instances, and surmise in very many more, never think their disappointment can have anything to do with the quality of the bandages employed, but that it is due to my having overstated the value and efficacy of the new method. Reports have been published in English medical journals of precisely the same failure of the process of cicatrization noticed at the Roosevelt Hospital, which has been due to the use of bandages reeking with carbolic acid. I wrote to the reporter, informing him of the cause of his partial failure; he procured bandages from my London agents, and results obtained from their use, with the very patients on whom the carbolized bandage had failed, speedily convinced him that my explanation was correct. I sent the same information, for the benefit of the European profession, to two of the most top-lofty of English medical journals. It was not published, and for this simple and sole reason, viz., that the manufacturers of the bandages which had been, at one stage of the manufacture, soaked in a strong solution of carbolic acid, to disguise the fetor of inferior "gum," and with a forlorn hope to prevent its rapid deterioration or "rotting," advertised very largely in the said high minded journals, and are the source of pleasant rills of income flowing into their coffers. It was, of course, in their estimation, far better that practitioners should be cheated, patients wronged and injured, and (but that is hardly worth mentioning) my method misrepresented and misjudged, than that the pleasant and mutually profitable relations between most "respectable" and wealthy though rascally dealers and these dignified journals should be in any way or degree disturbed, and, above all, in the interest of an unknown and insignificant trans-Atlantic doctor.

Without entering at all into a detailed account of the very numerous and interesting processes in the manufacture of pure rubber bandages, I will state that "Martin's bandages" *should* be, and those inspected by myself and sold by my agents *are*, made of the very finest selected extra price Para rubber, thoroughly cleaned, and losing from twenty to twenty-five per cent. thereby, rolled into coarse sheets, called "mats," and seasoned for from at least 6 to 12 months. In addition to this, literally nothing except the extreme minimum of pure sublimated sulphur, absolutely necessary with heat to effect the "curing," enters into the composition of the bandage. At a certain stage of manufacture, just before curing, the immense sheets of which my bandages are made are covered with an impalpably fine powder of French chalk. This, with a slight efflorescence of sulphur, produces the light grayish appearance of my bandages, but this is quite extraneous, and is entirely removed by a few days' wear. This purity of the material of the bandages is of the very highest importance, as is also another quality, viz., the most perfect possible smoothness. In previous writings I have not dwelt on this. I had never used any bandages except smooth ones; it never occurred to me that any one would make bandages to be applied directly to inflamed, irritable and ulcerated surfaces other than smooth. Since those publications, great numbers of bandages have been made (once, by a rascally manufacturer, for myself) deficient in smoothness, and the effects from the use of these have instructed me as to the necessity of cautioning the profession against any rubber bandage, if to be worn next to the skin, that is not, besides being pure, as above indicated, of the most perfect attainable smoothness. Very much against my original intention and wish, as the only way to save the innovation in treatment in which I am so deeply interested from misjudgment and more or less oblivion and neglect, I have become interested in the manufacture of rubber bandages. The manufacturer, a warm personal friend, and enthusiastic believer in my method, has been so kind as to give the matter an amount of his personal attention and supervision which, as a matter of business, can never be repaid; at every stage of the manufacture he gives me every facility for inspection, and every bandage made by him for me is stamped with a *fac simile* of my signature in carmine ink, and fully warranted to possess the most perfect durability and to be made of the very best material, in the very best possible way. These bandages, made in twelve different sizes, and of four different degrees of thickness, can be obtained from myself, or from either of my agents, in London and Boston, who are instructed to at once exchange any bandage thought to be defective in any way for a perfect one, or return the price, as the purchaser may elect.

I would gladly have avoided the trouble the supervision and care of this business gives me. I will at once drop it, so soon as the market is fully supplied, from other sources, with "Martin's bandages," such as alone I recommend, or have ever recommended. At present, I regard the continuance of the enterprise as absolutely necessary to my own reputation and that of the method I have fully and frankly offered to the profession and the public.

I offer no apology for the length of this letter. The physicians of the West largely employ "Martin's bandages." They were led to adopt the practice from hearing my remarks, at Chicago, in June, 1877, and from reading my paper, in your journal, in November, 1877, and it is nothing less than a duty for me to write, for their instruction, much contained in this communication.

If gentlemen rigidly and exactly following the directions I have given, fail to obtain results of treatment such as I have indicated and promised, let them first thoroughly and accurately ascertain whether the bandages they have used are such as alone I recommend. If they find that the bandages are all right, then let them "pitch into" me and my method; but if they use bandages such as are sold by the thousand, and adopt "dodges" and improvements on my method, they must see the injustice of reporting their results as results of Martin's method of using "Martin's bandage."

Henry A. Martin,
June 23, 1879. 27 Dudley St., Boston.

